# Post-marketing withdrawal of anti-obesity medicinal products because of adverse drug reactions: a systematic review

**DOI:** 10.1186/s12916-016-0735-y

**Published:** 2016-11-29

**Authors:** Igho J. Onakpoya, Carl J. Heneghan, Jeffrey K. Aronson

**Affiliations:** 1 Nuffield Department of Primary Care Health Sciences, University of Oxford, Centre for Evidence-Based Medicine, Gibson Building, Radcliffe Observatory Quarter, Oxford, OX2 6GG UK; 2 Nuffield Department of Primary Care Health Sciences, University of Oxford, Centre for Evidence-Based Medicine, Radcliffe Primary Care Building, Radcliffe Observatory Quarter, Oxford, UK

**Keywords:** Obesity, Drug withdrawal, Adverse drug reaction, Systematic review

## Abstract

**Background:**

We identified anti-obesity medications withdrawn since 1950 because of adverse drug reactions after regulatory approval, and examined the evidence used to support such withdrawals, investigated the mechanisms of the adverse reactions, and explored the trends over time.

**Methods:**

We conducted searches in PubMed, the World Health Organization database of drugs, the websites of drug regulatory authorities, and selected full texts, and we hand searched references in retrieved documents. We included anti-obesity medications that were withdrawn between 1950 and December 2015 and assessed the levels of evidence used for making withdrawal decisions using the Oxford Centre for Evidence-Based Medicine criteria.

**Results:**

We identified 25 anti-obesity medications withdrawn between 1964 and 2009; 23 of these were centrally acting, via monoamine neurotransmitters. Case reports were cited as evidence for withdrawal in 80% of instances. Psychiatric disturbances, cardiotoxicity (mainly attributable to re-uptake inhibitors), and drug abuse or dependence (mainly attributable to neurotransmitter releasing agents) together accounted for 83% of withdrawals. Deaths were reportedly associated with seven products (28%). In almost half of the cases, the withdrawals occurred within 2 years of the first report of an adverse reaction.

**Conclusions:**

Most of the drugs that affect monoamine neurotransmitters licensed for the treatment of obesity over the past 65 years have been withdrawn because of adverse reactions. The reasons for withdrawal raise concerns about the wisdom of using pharmacological agents that target monoamine neurotransmitters in managing obesity. Greater transparency in the assessment of harms from anti-obesity medications is therefore warranted.

**Electronic supplementary material:**

The online version of this article (doi:10.1186/s12916-016-0735-y) contains supplementary material, which is available to authorized users.

## Background

The prevalence of corpulence and obesity has more than tripled in the last decade [1], giving rise to substantially increased healthcare costs [2, 3]. According to the World Health Organization (WHO), corpulence and obesity are the fifth leading cause of mortality worldwide [4]. Strategies to reduce weight include diet and lifestyle adjustments, behavioural techniques, and drug therapy [5]; however, they have had little effect on the growing obesity epidemic.

Modern drug therapy for obesity treatment began in 1933 with the use of 2,4-dinitrophenol (2,4-DNP), a thermogenic agent, for weight reduction [6]. However, its use was associated with serious adverse reactions, and the US Food and Drug Administration (FDA) banned it in 1938 [7]. In the late 1930s, drugs acting on monoamine neurotransmitters, prescribed as appetite suppressants, were introduced [8]. One such drug, Hydrin (amphetamine), gained FDA approval under controversial circumstances, because the FDA’s then acting medical director served as a consultant to the Council on Pharmacy and Chemistry of the American Medical Association (AMA) [9]. The council later withdrew its approval of Hydrin, citing lack of evidence on harms [10]. This was followed by the development of other anti-obesity medications, mainly congeners of amphetamine and monoamine re-uptake inhibitors [11].

In recent years, there has been a growing focus on the development of novel therapies for the treatment of obesity [12], including drug combinations, a few of which have recently gained marketing approvals from regulatory authorities [13].

However, at the same time several anti-obesity products have been withdrawn from the market after approval because of adverse reactions [14]. To date, the patterns of and reasons for withdrawal of anti-obesity products have not been systematically analysed. Therefore, our objectives were to identify anti-obesity products withdrawn from the market because of adverse reactions, to examine the evidence used for withdrawals, determine the mechanism through which the adverse reactions occurred, and explore the patterns in withdrawal over time.

## Methods

### Search strategy

We searched for anti-obesity medications that had been withdrawn from the market after regulatory approval because of adverse drug reactions between 1950 and December 2015 from the following sources:

- WHO database of Consolidated List of Products whose consumption and/or sale have been banned, withdrawn, severely restricted, or not approved by governments (issues 6, 8, 12, and 14)
- Regulatory news sections of WHO Drug Information (volumes 1–29)
- WHO Pharmaceuticals Newsletter (1997–2015)
- UK Medicines and Healthcare products Regulatory Agency (MHRA) website
- US FDA website
- Database of withdrawn drugs of the European Medicines Agency (EMA)

For each withdrawn anti-obesity medicine identified, we searched the following sources for the first reported adverse drug reaction: PubMed, Medline, Google Scholar, *Meyler’s Side Effects of Drugs* and the *Side Effects of Drugs Annuals* 1–36, and *Stephens' Detection of New Adverse Drug Reactions*, 5th edition. Search terms used included “anti-obesity”, “withdrawal”, “fatal*”, “adverse reaction”, “adverse event”, “toxicity”, “voluntary recall”, “suspension”, “prohibition”, “banned”, “remov*”, “revoke*”, and “discontinued”. (See Additional file 1 for extended search lists of websites and other sources accessed, together with search strategies used for searching scientific databases.) If we could not find information for an anti-obesity medication when using its chemical name for searches, we used trade or code names. We also hand searched references in retrieved full texts for earlier reports of adverse reactions. If an article contained information about an earlier reported date, that date was chosen as the first adverse reaction date. If an anti-obesity medicine was withdrawn because of two or more adverse reactions, we used the date of the first reported reaction.

### Inclusion/exclusion criteria

To be included in the review, an anti-obesity medicinal product must have been withdrawn from the market because of reports of suspected adverse reaction(s) or problems related to hazards or harms. We excluded anti-obesity medicines for which there was documented regulatory evidence that they had been voluntarily withdrawn by marketing authorization holders (MAHs) solely for commercial reasons. We also excluded herbal, non-human, and non-prescription medicines (i.e. medicines that were not approved via the conventional regulatory pathway for drug licensing) used as weight loss products.

### Assessing the types of evidence

We documented the highest level of available evidence before the year of first withdrawal of anti-obesity medicines, based on the Oxford Centre for Evidence-Based Medicine (OCEBM) criteria [15], which rank the levels of evidence of harms as follows: Level 1, systematic review (highest); Level 2, randomized clinical trial; Level 3, non-randomized cohort or follow-up studies; Level 4, case-series or case-control studies; and Level 5, mechanism-based reasoning (lowest). One reviewer (IJO) documented the levels of evidence, which were independently verified by a second reviewer (JKA). Disagreements were resolved through discussion.

### Data extraction

For each withdrawn anti-obesity product, we extracted data on the marketing authorization or launch date (or the date of first recorded use); the drug class, mechanism of action, and therapeutic indication(s) [16]; the year an adverse reaction related to the reason for withdrawal was first reported; the year of first withdrawal; the country or countries of withdrawal; and the reported mechanism by which the drug caused the adverse reaction. One reviewer (IJO) extracted the data, and a second reviewer (JKA) verified them independently. Discrepancies were resolved by discussion.

### Statistical analyses

We used summary tables to document the intervals between launch year and the year of first report of an adverse drug reaction, the interval between launch year and the year of first withdrawal, and the interval between the first report of an adverse drug reaction and the year of first withdrawal. Because these intervals were skewed, we used medians and interquartile ranges (IQRs). We also compared intervals to reports of adverse reactions and withdrawals between products based on their mechanistic actions. Fisher’s exact test was used to test for differences in the proportions of withdrawals based on the pharmacological mechanisms of action; a *P* value of <0.05 was considered statistically significant.

## Results

We identified 47 withdrawn medicinal products used for treating obesity (Additional file 2). We excluded 11 products because they were plant-based “herbal” products and another 11 because they were discontinued before being granted regulatory approval. This left 25 products for inclusion. Two of the products, fenfluramine and dexfenfluramine, were withdrawn worldwide. Of the remaining 23 products, 19 were withdrawn in Europe, 11 in Asia, 9 in South America, 8 in North America, 4 in Africa, and 1 in Australasia (Table 1).

**Table 1:** List of anti-obesity drugs withdrawn from the market because of adverse drug reactions

Medicinal product	Primary mechanism of action	Therapeutic indication	Launch date	Year of first ADR report	Year first withdrawn	Countries withdrawn	Primary reason for withdrawal	Level of evidence^a^
Amfepramone (diethylpropion)^b^	SNDRA	Obesity	1957	1974	1975	Turkey, Sweden, Oman, UAE, Norway, Venezuela, EU, France, UK, Brazil	Cardiotoxicity	4
Amphetamine	SNDRA	Obesity, narcolepsy	1939	1957	1973	USA, UAE, Turkey, Oman, Malaysia, Nigeria	Drug abuse and dependence	4
Aminorex fumarate^c^	SRI	Obesity	1962	1967	1967	Germany, Venezuela, Switzerland, Austria	Cardiotoxicity	4
Benfluorex^c^	SRI	Obesity, diabetes	1976	2003	2009	Europe	Cardiotoxicity	3
Chlorphentermine	SRI	Obesity	1962		1969	Germany, Venezuela	Cardiotoxicity	5
Clobenzorex	SNDRA	Obesity	1966	1986	2000	Mauritius, USA, Oman	Drug abuse, psychiatric	4
Cloforex	SRI	Obesity	1965	1967	1967	Germany, Sweden, Venezuela	Cardiotoxicity	4
Cyclovalone + retinol + tiratricol	Bile acid secretion	Hyperlipidemia, dyspepsia, obesity	1964	1984	1988	France	Liver toxicity	4
Dexfenfluramine	SRI	Obesity	1995	1995	1997	Worldwide	Cardiotoxicity	4
Fenbutrazate	NDRA	Obesity	1957	1963	1969	Europe	Drug abuse, psychiatric	2
Fenfluramine^c^	SRI	Obesity	1973	1981	1997	Worldwide	Cardiotoxicity	3
Fenproporex (perphoxene)	NRA	Obesity, narcolepsy, ADHD	1966	1997	1999	Europe, Brazil	Drug abuse, psychiatric	4
Iodinated casein strophanthin	Thyroxine analogue	Obesity	1944	1964	1964	USA	Endocrine, metabolism	4
Levamphetamine	SNDRA	Obesity	1944	1954	1973	USA, Oman, UAE	Drug abuse and dependence	4
Mazindol	NRDA	Obesity	1970	1980	1987	Oman, Brazil	Drug abuse, psychiatric (interaction with lithium)	4
Mefenorex (methylphenethylamine)	SNDRA	Obesity	1966	1995	1999	Europe, Oman	Drug abuse, psychiatric	4
Methamphetamine (desoxyephedrine)^c^	SNDRA	ADHD, obesity	1944	1971	1973	USA, Turkey, Oman, Nigeria	Drug abuse, drug dependence	4
Phendimetrazine	NDRA		1961	1979	1982	Turkey	Drug abuse	4
Phenmetrazine	NDRA	Obesity	1956	1959	1982	Turkey, Oman, Nigeria	Drug abuse	4
Phentermine^b,c^	NDRA	Obesity	1959	1964	1981	Sweden, UAE, Mauritius, Turkey, Oman, UK, Venezuela	Drug abuse	4
Phenylpropanolamine (norpseudoephedrine)	NDRA	Nasal decongestion, obesity	1947	1985	1987	Germany, Brazil, Malaysia, Singapore, USA, Oman, Canada, Cuba, India	Hemorrhagic stroke	4
Pipradrol	NDRI	Obesity, narcolepsy, ADHD	1953	1968	1982	USA, Turkey, Denmark, Venezuela	Drug abuse	4
Pyrovalerone	NDRA	Obesity, chronic fatigue syndrome	1974	1975	1979	France	Drug abuse	4
Rimonabant^c^	CB_1_ antagonist/inverse agonist	Obesity	2006	2006	2007	Europe, India	Psychiatric	1
Sibutramine^c^	SNRI	Obesity	2001	2002	2002	EU; 4 Asian countries; Australia; Canada; Mexico; New Zealand; USA	Cardiotoxicity, psychiatric	4

*ADHD* attention deficit hyperactivity disorder, *ADR* adverse drug reaction, *CB*_*1*_ cannabinoid 1 (receptors), *EU* European Union, *NRA* norepinephrine releasing agent, *NDRA* norepinephrine-dopamine releasing agent, *NDRI* norepinephrine-dopamine re-uptake inhibitor, *SNDRA* serotonin-norepinephrine-dopamine releasing agent, SNRI serotonin-norepinephrine re-uptake inhibitor, SRI serotonin re-uptake inhibitor, *UAE* United Arab Emirates

^a^Based on the Oxford Centre for Evidence-Based Medicine Levels of Evidence [17]. Level 1: systematic review of randomized trials, systematic review of nested case-control studies, Level 2: individual randomized trial or (exceptionally) observational study with dramatic effect; Level 3: non-randomized controlled cohort/follow-up study (post-marketing surveillance); Level 4: case-series, case-control, or historically controlled studies; Level 5: mechanism-based reasoning

^b^Re-introduced in the EU based on long-standing legal action unrelated to either new safety or new efficacy information

^c^Reported to have caused deaths

Of the 25 withdrawn products, 22 (92%) were appetite suppressants acting on monoamine neurotransmitters (Table 1). Their primary mechanisms of action were through central effects on adrenoceptors and dopamine receptors (8); adrenoceptors, dopamine receptors, and serotonin receptors (5); serotonin receptors only (6); adrenoceptors only (1); adrenoceptors and serotonin receptors (1); and serotonin and dopamine receptors (1). One drug acted on cannabinoid receptors, one by stimulating bile acid secretion, and one by attachment to thyroid hormone binding proteins.

Case reports (Level 4 evidence) were cited as evidence for withdrawal in 20 instances (80%), and Level 3 evidence in two instances (8%) (Table 1). The withdrawal of one product, chlorphentermine, was based on evidence from animal studies. Cardiotoxicity accounted for 8 withdrawals (32%) and psychiatric adverse reactions for 7 (28%). Drug abuse or dependence was cited in more than half of the withdrawals (13 cases, 52%), and drug-attributed deaths were associated with the withdrawal of 7 products (28%).

Of the 23 withdrawn products acting via monoamine neurotransmitters, 8 were re-uptake inhibitors and 14 were releasing agents (Table 1). One product, rimonabant, was an antagonist and inverse agonist at cannabinoid receptors. The releasing agents were significantly more likely to be withdrawn because of drug abuse compared with the re-uptake inhibitors (*P* = 0.002); the re-uptake inhibitors were significantly more likely to be withdrawn because of cardiovascular adverse reactions compared with the releasing agents (*P* = 0.0004). Psychiatric adverse reactions were not significantly different between the two groups of agents.

The longest interval between launch and the first report of an adverse drug reaction was 38 years (norpseudoephedrine), while the shortest interval was less than 1 year (rimonabant). The median interval between launch and first report of an adverse drug reaction was 10 years (IQR = 3 to 20 years). In 24% of cases, first reports of the adverse drug reactions in the literature occurred within 2 years. Overall, there was a shortening in the interval between launch and first published reports of adverse drug reactions. The interval between launch and first reports of adverse reactions was considerably shorter with the re-uptake inhibitors than with the releasing agents: 5.5 years (IQR = 1.3 to 13 years) versus 18 years (IQR = 5.8 to 28 years); see Table 2. The median interval to first reports of adverse reactions was considerably shorter when there was cardiotoxicity compared with drug abuse: 5.5 years (IQR = 1.3 to 15 years) versus 15 years (IQR = 5.5 to 24 years).

**Table 2:** Summary comparison of post-marketing withdrawal patterns of centrally acting anti-obesity medicinal products

Primary mode of action at receptor ending	Primary reason for withdrawal	Median interval between launch and first ADR report	Median interval between first ADR report and withdrawal	Statistical comparisons^a^
Neurotransmitter re-uptake inhibition (*n* = 8)	Cardiotoxicity: *n* = 7 Drug misuse: *n* = 1	5.5 years (IQR = 1.3 to 13.3 years	1 year (IQR = 0 to 12 years)	Withdrawal due to cardiotoxicity significantly more likely vs neurotransmitter releasers (*P* = 0.0004)
Neurotransmitter release (*n* = 14)	Cardiotoxicity: *n* = 1 Drug abuse or dependence: *n* = 12 Hemorrhagic stroke: *n* = 1	17.5 years (IQR = 5.8 to 28 years)	5 years (IQR = 2 to 16.3 years)	Withdrawal due to drug misuse significantly more likely vs neurotransmitter re-uptake inhibitors (*P* = 0.002)

One centrally acting product, rimonabant, has been excluded from the comparisons because it is an antagonist and inverse agonist at cannabinoid C1 receptors

^a^Using Fisher’s exact test; there was no significant difference between groups for proportion of psychiatric disturbances (*P* = 1.000)

*ADR* adverse drug reaction, *IQR* interquartile range

**Fig. 1:**
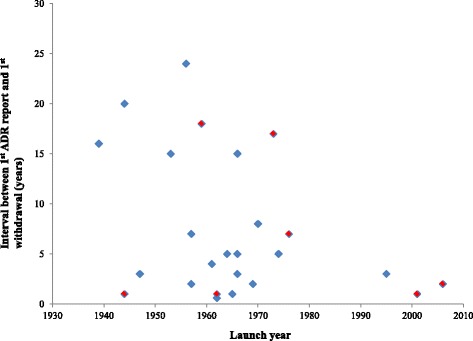
Interval between first ADR reports and first withdrawals. The red boxes indicate products to which deaths were attributed

The longest interval between first report and first withdrawal was 23 years (phenmetrazine), while the shortest interval was less than 1 year in three cases (aminorex fumarate, cloforex, and iodinated casein; Table 1). The median interval between first report and first withdrawal was 11 years (IQR = 0 to 23 years). Figure 1 shows that the more recent the launch year, the faster the product was withdrawn from the market following adverse reaction reports. In 48% of cases, withdrawals occurred within 2 years of the first adverse drug reaction (ADR) reports. When deaths were attributed to the products, the median interval to withdrawal was 1 year. The median time to withdrawal following reports of adverse reactions was shorter with the re-uptake inhibitors than the releasing agents: 1 year (IQR = 0 to 12 years) versus 5 years (IQR = 2 to 16 years); Table 2. The median time to withdrawal following reports of adverse reactions was considerably longer with drugs that caused abuse or dependence than with drugs that caused cardiotoxicity: 7 years (IQR = 3.5 to 17 years) versus 0.5 year (IQR = 0 to 5 years).

## Discussion

We have identified 25 anti-obesity medications that were withdrawn after marketing between 1964 and 2009, 23 of which had centrally acting mechanisms via monoamine neurotransmitters. The evidence for withdrawal in about 80% of cases was anecdotal reports. Cardiovascular and psychiatric adverse drug reactions and drug abuse and dependence together accounted for 83% of the withdrawals. In 28% of cases, deaths were attributed to the products. Among the centrally acting agents, re-uptake inhibitors were more likely to be withdrawn because of cardiotoxicity, while the neurotransmitter releasers were more likely to be withdrawn because of drug abuse and dependence. When compared with our overall corpus of 462 medicinal products withdrawn from the market after 1950 [17], there was no significant difference in the proportion of case reports being used as evidence for withdrawal. The median interval to first reports of adverse reactions and first withdrawals was longer with the anti-obesity products (11 versus 3 years), but this is likely due to the smaller proportion of anti-obesity product approvals after 1976 compared with the overall data (the median interval to withdrawal for the products withdrawn since 1995 was 3 years). Furthermore, the intervals to withdrawal with more recent product launch are also consistent with the trend observed in the overall corpus.

The use of case reports as evidence for making withdrawal decisions in a majority of instances corroborates our previous findings that formal studies are seldom conducted when adverse drug reactions are suspected [18]. In addition, the fact that most of the withdrawn products were centrally acting anorectics suggests that use of this class of products in the treatment of obesity is associated with a negative benefit-to-harm balance over time (see Table 3 and Additional file 3). Furthermore, the discrepancy in withdrawal patterns across regulatory authorities indicates a lack of uniformity in harms assessment by different drug regulators, and also contributes to discrepancies in patterns of marketing authorizations of this class of products.

**Table 3:** Profile of centrally acting anti-obesity products withdrawn because of associated deaths over the last 50 years

Aminorex • First introduced in 1962 • Became available within 3 years of introduction as an over-the-counter weight loss pill • Between 1965 and 1972, there was an alarming increase in the incidence of primary pulmonary hypertension [52] • The pulmonary hypertension epidemic ended in 1972 following the withdrawal of aminorex from the market	
Benfluorex • Approved in 1976 as an add-on treatment in obese patients with diabetes mellitus • Cases of valvulopathy attributed to its use began to appear from 2003 • Was withdrawn in 2009 following an epidemic of valvulopathy attributed to its use • Several deaths reported • To date, more than 3000 hospitalizations and at least 1300 deaths attributed to its use in France alone [53]	
Fenfluramine • First approved in in 1973 • Reports of pulmonary hypertension first appeared in 1981 • Several other case reports subsequently published • Epidemiological studies showed an association between fenfluramine and pulmonary hypertension [54] • Withdrawn worldwide in 1997	
Methamphetamine (desoxyephedrine) • First introduced in 1944 • Within 10 years of its introduction, cases of its misuse had been reported • By the early 1970s, reports of its abuse as an anorectic were reported [55] • Was withdrawn in the USA and other countries in 1973 • Several cases of cardiac abnormalities related to its abuse have subsequently been reported	
Phentermine • First approved in 1959 • Several cases of lung phospholipidosis in animals and humans reported thereafter • Reports of death began to appear in 1974 [56] • Withdrawn from most countries where it was marketed in 1981 • Still available for short-term management of obesity in the USA	
Rimonabant • Approved in Europe in 2006 for obesity treatment • Within 1 year of approval, concerns were expressed about the risk of depression and suicide associated with its use [57] • Five deaths attributed to its use in the UK [58] • Was withdrawn in 2007	
Sibutramine • Approved in 1997 in the USA and Europe in 2001 • Within a year of its European approval, serious cardiovascular adverse reactions were reported, resulting in temporary withdrawal in Italy [59] • Several cases of severe cardiovascular adverse reactions, including deaths, were subsequently reported [60] • Withdrawn in Europe and USA in 2010	

Fishman AP. 1999 [52]

Fournier A, Zureik M. 2012 [53]

Connolly HM, Crary JL, McGoon MD, Hensrud DD, Edwards BS, Edwards WD, Schaff HV. 1997 [54]

Ladewig D, Battegay R. 1971 [55]

Price K. 1974 [56]

Gadde KM. 2006 [57]

World Health Organization. 2008 [58]

Anonymous. 2002 [59]

Wooltorton E. 2002 [60]

That the delays to reports of adverse reactions with time were shortened, albeit inconsistently, suggests an influence of better methods for detection of adverse drug reactions, improved transparency in the report of adverse drug events by trials investigators, or a combination of the two. However, the speed with which withdrawals occurred following reports of suspected adverse reactions with more recently launched drugs arouses suspicion of selective reporting of harms in the pre-marketing phase. The shorter intervals to withdrawals when deaths are reported suggest that regulatory authorities are quicker at making withdrawal decisions in these circumstances.

### Comparison with existing literature

Our findings are consistent with other published reports. A previous review reported that most anti-obesity treatments have been withdrawn from the market because of concerns about adverse reactions [14]. Two other reviews concluded that cardiovascular and psychiatric adverse reactions are major concerns with psychoactive anti-obesity drugs that have recently gained marketing approval from licensing authorities [19, 20]. A recent analysis of serious adverse reactions reported to the EudraVigilance database also showed that cardiac and psychiatric disorders were the most common adverse reactions attributed to anti-obesity medicinal products [21]. The authors of another report concluded that lower doses of multiple chemical entities targeting different mechanistic pathways should be a priority in future drug development [1].

### Strengths and limitations

To our knowledge, this is the first review that has systematically identified anti-obesity medicinal products withdrawn from the market because of adverse drug reactions. We comprehensively searched various sources of information in order to identify anti-obesity medicinal products that have been withdrawn, we accounted for the levels of evidence used in making withdrawal decisions, extracted data on the intervals between launch, adverse reactions reports, and withdrawals, and explored the trends in withdrawal over time. We also used statistical methods to explore and compare the patterns of withdrawal. However, we recognize some limitations. We may not have identified all countries in which products were withdrawn, and we do not have complete information about where all the products were marketed; indeed it is possible that some products were withdrawn in a few countries because those were the only places where they were marketed. In addition, we do not have information on the time lapse between the actual occurrence of an adverse reaction and the date the reaction appeared in the published literature, though we do not consider that such delays would have significantly influenced our findings. Finally, the intervals between launch, first adverse reaction reports, and first withdrawals were computed irrespective of where products were first introduced or where adverse reactions were first observed; however, some products were withdrawn in some countries because of adverse reactions reported in others irrespective of whether the product was first marketed in such countries.

### Implications for drug therapy

Apart from two products (cyclovalone and iodinated casein), the products identified in the review had actions involving 5-hydroxytryptamine (5HT, serotonin). While agonist activity at 5HT_2A_ receptors can cause psychiatric dysfunction [22], stimulation of 5HT_2B_ receptors is associated with cardiac abnormalities, largely valvulopathies [23].The unfavourable benefit-to-harm balance of this class of products therefore appears to have led to the development of new chemical entities that target other mechanistic pathways. These include leptin sensitizers, glucagon-like peptide 1 (GLP-1) receptor agonists, islet amyloid polypeptide (IAPP), and neuropeptide Y [24]. Of note, several medicinal products with similar mechanisms of action to the withdrawn psychotropic anti-obesity medications have been successfully used for treating other medical conditions, and they have not been withdrawn from the market. This may be because in those conditions their mechanisms of action are specifically targeted against abnormal pathways. For example, several analogues of amphetamine are available for treating attention deficit hyperactivity disorder (ADHD) and narcolepsy [25, 26].

There were no new approvals of anti-obesity medications during the 20 years from 1976 to 1995. It is unclear what caused this hiatus; however, we observed a steep increase in the number of scientific publications relating obesity prevalence and treatment after the hiatus (PubMed trend; Additional file 4). The resurgence of new approval applications after the hiatus was not associated with a major shift in the development of drugs that affect neurotransmitters, but it is notable that no new drugs that cause neurotransmitter release have been marketed since 1974. This is consistent with the fact that of the nine medicinal products currently available for obesity management, all six (78%) that exert their weight reducing actions through centrally acting mechanisms act via neurotransmitter release, rather than inhibition of re-uptake (see Table 4). This suggests that drug developers have largely abandoned the use of chemical entities that inhibit re-uptake in favour of releasing agents. However, the lack of long-term data on the safety of these products, especially with the more recent approvals, is of great concern. Indeed, psychiatric and cardiovascular adverse events have been reported as major concerns of recently approved products with centrally acting mechanisms [19].

**Table 4:** Anti-obesity drugs currently approved for use in at least one country

Brand name	Active ingredient	Mechanism of action	Year approved
Adipex-P, Ionamin	Phentermine	Precise mechanism unknown: norepinephrine-dopamine releasing agent	1959
Alli, Xenical^a^	Orlistat	Inhibits gastric and pancreatic lipases	1999
Belviq^a^	Lorcaserin	Selective 5-HT_2C_ receptor agonist	2012
Contrave, Mysimba^a^	Naltrexone + bupropion	Bupropion: re-uptake inhibitor and releasing agent of norepinephrine and a nicotinic acetylcholine receptor antagonist; naltrexone augments bupropion’s activation of proopiomelanocortin (POMC)	2014
Didrex	Benzphetamine	Precise mechanism unknown: norepinephrine-dopamine releasing agent	1960
Obezine	Phendimetrazine	Precise mechanism unknown: norepinephrine-dopamine releasing agent	1961
Qsymia, Qnexa^a^	Phentermine + topiramate	The precise mechanism of action for both drugs is unknown: phentermine is a norepinephrine-dopamine releasing agent; topiramate augments gamma-aminobutyrate (GABA), inhibits α-amino-3-hydroxy-5-methyl-4-isoxazolepropionic acid (AMPA)/kainate glutamate receptors, and inhibits carbonic anhydrase	2012
Saxenda, Victoza^a^	Liraglutide	Glucagon-like peptide-1 receptor agonist	2014
Tenuate Dospan	Diethylpropion	Precise mechanism unknown: norepinephrine-dopamine releasing agent	1959

^a^Approved for long-term treatment

Modest reduction in body weight has beneficial effects on short-term (≤1 year) cardiovascular profile [27]. However, whether the weight losses generated by anti-obesity medicinal products have beneficial effects on long-term cardiovascular outcome is unclear, largely because the follow-up duration of most clinical trials is not long enough to assess these benefits beyond the weight losses achieved [28]; indeed, one large long-term study (*n* = 5145; median follow-up of 9.6 years) showed that weight reduction did not reduce the risk of adverse cardiovascular events in obese and overweight adults with type 2 diabetes [29]. In addition, the benefit of aggressive weight reduction in overweight and obese patients with cardiovascular decompensation (e.g. patients with heart failure) has been questioned, because this group of patients survives for a longer period at higher body weights [30]. Furthermore, more than one-third of the centrally acting products included in our review were withdrawn because their use was associated with adverse cardiovascular outcomes that are the health risks the use of the products were intended to reduce — an “obesity treatment paradox”.

### Implications for future harms assessment and policy

Although there have been revisions to regulatory guidance for the assessment of harms in anti-obesity drug trials [31, 32], there are uncertainties about the extent to which these guidelines are applied when assessing trial results for new weight loss products whose actions are through central mechanisms. For example, lorcaserin, a 5HT_2C_ receptor agonist, was approved by the FDA based on the results of three pivotal trials [33–35], with discontinuation rates of 45%, 50%, and 34%, with mean differences in weight loss of only 3–3.6%. Although two of the three trials met the FDA guidance for effectiveness (“The proportion of subjects who lose greater than or equal to 5 percent of baseline body weight in the active-product group is at least 35%, is approximately double the proportion in the placebo-treated group, and the difference between groups is statistically significant”) [32], the analysis was based on the per-protocol population for completers. Furthermore, the trials failed to meet the FDA’s guidance for products for weight management, which sets clinical significance at 1 year as 5% weight loss with at least 80% power. Indeed, the FDA noted that baseline psychiatric history was limited in the clinical trials, discontinuations owing to nervous system and psychiatric disorders were higher with lorcaserin than with placebo, and the trials were not powered to detect cardiovascular adverse events [36]; these same concerns influenced the decision of the EMA in declining the product’s approval application [37]. In addition, transcripts from the FDA showed that although members of the voting panel approved the product, many had concerns about cardiovascular harms [38]; it is of further concern that the post-marketing surveillance of the product is behind schedule [39].

Similarly, the approval of naltrexone plus bupropion (Contrave, Mysimba) by the FDA and EMA has been questioned by some authors because of the potential for severe harms that appear to outweigh the benefits [40, 41]. In a recent large randomized trial there was a significantly increased risk of psychiatric disorders, and the cardiovascular profile of the product is uncertain [42]. These questionable approvals by regulatory authorities lend credence to the argument that an independent group of experts should be given responsibility for assessing harms [43]. The re-introduction of amfepramone (diethylpropion) and phentermine in Europe, based on a long-standing legal action, unrelated to new information on benefits or harms, is another case in point [44, 45].

The herbal weight loss medication *Ephedra spp.*, which has agonist activity at 5HT and dopamine receptors and showed evidence of beneficial effects on body weight, was banned by the FDA in 2004 because of an increased risk of cardiovascular and psychiatric adverse reactions [46, 47]. That regulatory authorities removed this product from the market yet granted its “conventional analogues” marketing licences arouses suspicion of regulatory bias [48].

In addition to the above, several of the products that have been withdrawn by regulatory authorities are now marketed as non-prescription formulations, alone, or in combination over the counter, or via the Internet [49]. Indeed, there have been many reports of suspected adverse reactions attributed to such products [7, 50, 51]. There should be tougher legislative action against the promoting and marketing of such products.

## Conclusions

We have identified 25 anti-obesity medications withdrawn after marketing over the past 60 years, 23 of which were centrally acting anorectics. Psychiatric disturbances, cardiotoxicity, or drug abuse and dependence accounted for more than 80% of withdrawals. Centrally acting products that caused release of monoamines were significantly more likely to be withdrawn because of adverse cardiovascular reactions, while monoamine re-uptake inhibitors were more likely to be withdrawn because of drug abuse and dependence. Greater transparency in the reporting of the harms by trial investigators should be encouraged. Individuals with vested interests could be excluded from panels that consider new drug approval applications.

## Additional files

Additional file 1: Extended search lists of accessed sources used to identify anti-obesity medicinal products withdrawn from the market because of adverse drug reactions. (PDF 146 kb)
Additional file 2: Databases and selected texts used to identify withdrawn anti-obesity medications, their launch dates, dates of first adverse drug reaction reports, and withdrawals. (PDF 36 kb)
Additional file 3: Profiles of 23 centrally acting anti-obesity medicinal products withdrawn over the last 50 years because of adverse drug reactions. (PDF 246 kb)
Additional file 4: Publication trends* for scientific articles related to obesity prevalence and treatments over time. (PDF 16 kb)

## Abbreviations

2,4-DNP: 2,4-dinitrophenol
5HT: 5-hydroxytryptamine
ADHD: Attention deficit hyperactivity disorder
AMA: American Medical Association
EMA: European Medicines Agency
FDA: United States Food and Drug Administration
GLP-1: Glucagon-like peptide 1
IAPP: Islet amyloid polypeptide
IQR: Interquartile range
MAH: Marketing authorization holder
MHRA: UK Medicines and Healthcare products Regulatory Agency
WHO: World Health Organization
