# Supplementary material for: Post-marketing withdrawal of anti-obesity medicinal products because of adverse drug reactions: a systematic review
Source: BMC Med. 2016 Nov 29;14:191. doi: 10.1186/s12916-016-0735-y (PMC5126837; doi:10.1186/s12916-016-0735-y)
Supplement: Additional file 1: — Extended search lists of accessed sources used to identify anti-obesity medicinal products withdrawn from the market because of adverse drug reactions. (PDF 146 kb) [file 12916_2016_735_MOESM1_ESM.pdf]

Web Appendix 1: Extended search lists of accessed sources used to identify anti-obesity medicinal products withdrawn from the market because of adverse drug reactions.

The United Nations (UN) Consolidated List of Products whose consumption and/or sale have been banned, withdrawn, severely restricted, or not approved by governments: Issue 6 (pgs. 5-197); Issue 8 (pgs. 30-264); Issue 12 (pgs. 31-203); and Issue 14 (pgs. 3-26)

W.H.O. Drug Information: Regulatory Matters Section

2015: Vol. 29, Nos 1-4
2014: Vol. 28, Nos 1-4
2013: Vol. 27, Nos 1-4
2012: Vol. 26, Nos 1-4
2011: Vol. 25, Nos 1-4
2010: Vol. 24, Nos 1-4
2009: Vol. 23, Nos 1-4
2008: Vol. 22, Nos 1-4
2007: Vol. 21, Nos 1-4
2006: Vol. 20, Nos 1-4
2005: Vol. 19, Nos 1-4
2004: Vol. 18, Nos 1-4
2003: Vol. 17, Nos 1-4
2002: Vol. 16, Nos 1-2
2001: Vol. 15, Nos 1-4
2000: Vol. 14, Nos 1-4
1999: Vol. 13, Nos 1-4
1998: Vol. 12, Nos 1-4
1997: Vol. 11, Nos 1-4
1996: Vol. 10, Nos 1-4
1995: Vol. 9, Nos 1-4
1994: Vol. 8, Nos 1-4
1993: Vol. 7, Nos 1-2
1992: Vol. 6, Nos 1-4
1991: Vol. 5, Nos 1-3
1990: Vol. 4, Nos 1-4
1989: Vol. 3, Nos 1-4

1988: Vol. 2, Nos 1-4

1987: Vol. 1, Nos 1-3

W.H.O. Pharmaceuticals Newsletters: Regulatory Matters Section

2015: Newsletter Nos. 1-6

2014: Newsletter Nos. 1-6

2013: Newsletter Nos. 1-6

2012: Newsletter Nos. 1-6

2011: Newsletter Nos. 1-6

2010: Newsletter Nos. 1-6

2009: Newsletter Nos. 1-5

2008: Newsletter Nos. 1-6

2007: Newsletter Nos. 1-6

2006: Newsletter Nos. 1-6

2005: Newsletter Nos. 1-5

2004: Newsletter Nos. 1-6

2003: Newsletter Nos. 1-5

2002: Newsletter Nos. 1-4

2001: Newsletter Nos. 1-3

2000: Newsletter Nos. 1-4

1999: Newsletter Nos. 1-12

1998: Newsletter Nos. 1-12

1997: Newsletter Nos. 1-12

European Medicines Agency (EMA)

Find Medicine > Human Medicines > Browse by therapeutic area > Diseases > Nutritional and Metabolic Diseases > Nutrition Disorders > Overnutrition > Obesity > “Include: withdrawn post-approval, suspended”

Find Medicine > Human Medicines > Referrals > Browse by topic > Safety issues > “Include: Opinion provided by Committee for Medicinal Products for Human Use, European Commission final decision”

US Food and Drug Administration (FDA)

Home > Safety > Recalls, Market Withdrawals, & Safety Alerts > Archive for Recalls, Market Withdrawals & Safety Alerts

The UK Medicines and Healthcare products Regulatory Agency (MHRA)
Home > Drug safety update > Therapeutic area > Nutrition and Dietetics

Stephens' Detection of New Adverse Drug Reactions, 5th edition, 2004 (pgs. 667-702)

Pharmaceutical Manufacturing Manual, 3rd edition, 2006

The Merck Index, 15th edition, 2013

Meyler's Side Effects of Psychiatric Drugs 1st edition, 2009

Meyler's Side Effects of Cardiovascular Drugs 1st edition, 2009

Medline search strategy
"[Anti-obesity drug name]" AND "toxicity"
"[Anti-obesity drug name]" AND "adverse"
"[Anti-obesity drug name]" AND "side effect"
"[Anti-obesity drug name]" AND "poison"
"[Anti-obesity drug name]" AND "fatal*"
"[Anti-obesity drug name]" AND "fatality"
"[Anti-obesity drug name]" AND "death"
"[Anti-obesity drug name]" AND "withdrawal"
"[Anti-obesity drug name]" AND "withdrawn"
"[Anti-obesity drug name]" AND "recall"
"[Anti-obesity drug name]" AND "voluntary recall"
"[Anti-obesity drug name]" AND "banned"
"[Anti-obesity drug name]" AND "prohibited"
"[Anti-obesity drug name]" AND "remov*"
"[Anti-obesity drug name]" AND "discontinued"
"[Anti-obesity drug name]" AND "refus*"
[Sort by Publication date]

Google Scholar search strategy
Find articles with all of the words [anti-obesity drug name] > with the exact phrase [adverse or toxic or side effect or fatal or death or withdrawn or withdrawal or recall or banned or prohibited or discontinued or suspended] > Return articles dated between 1950-2015
