# Supplementary material for: Post-marketing withdrawal of anti-obesity medicinal products because of adverse drug reactions: a systematic review
Source: BMC Med. 2016 Nov 29;14:191. doi: 10.1186/s12916-016-0735-y (PMC5126837; doi:10.1186/s12916-016-0735-y)

Web appendix 2: Databases and selected texts used to identify withdrawn anti-obesity medications, their launch dates, dates of first adverse drug reaction reports and withdrawals

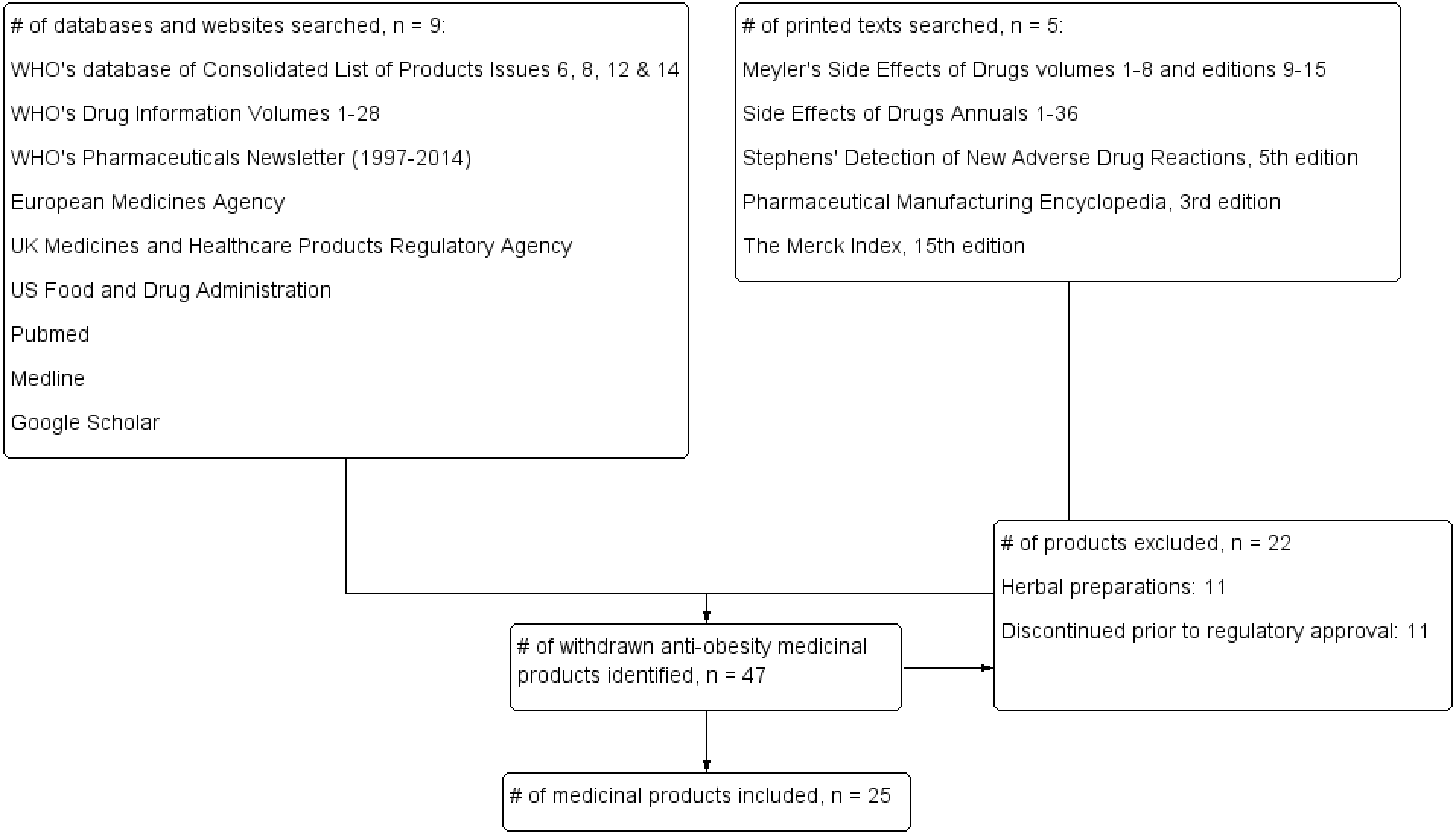

Supplement: Additional file 2: — Databases and selected texts used to identify withdrawn anti-obesity medications, their launch dates, dates of first adverse drug reaction reports, and withdrawals. (PDF 36 kb) [file 12916_2016_735_MOESM2_ESM.pdf]
