# Supplementary material for: Post-marketing withdrawal of anti-obesity medicinal products because of adverse drug reactions: a systematic review
Source: BMC Med. 2016 Nov 29;14:191. doi: 10.1186/s12916-016-0735-y (PMC5126837; doi:10.1186/s12916-016-0735-y)
Supplement: Additional file 3: — Profiles of 23 centrally acting anti-obesity medicinal products withdrawn over the last 50 years because of adverse drug reactions. (PDF 246 kb) [file 12916_2016_735_MOESM3_ESM.pdf]

## Web appendix 3: Profiles of 23 centrally-acting anti-obesity medicinal products withdrawn over the last 50 years because of adverse drug reactions

**Amfepramone**

- Mechanism of action: serotonin-norepinephrine-dopamine releasing agent
- Introduced as an anti-obesity agent in 1957
- Pickwickian syndrome attributed to its use first reported in 1974 [1]
- Withdrawn in several countries in 1974
- Fatal case of primary pulmonary arterial hypertension attributed to its use reported in the UK in 1995 [2]
- Reintroduced in the UK in 2002 with restrictions

**Amfetamine**

- Mechanism of action: serotonin-norepinephrine-dopamine releasing agent
- Initially used for narcolepsy
- Observed that patients with narcolepsy lost weight while taking amfetamine
- Approved in 1939 as an obesity treatment when the results of a trial showed that it was effective [3]
- Over dosage with its use as an anorectic first reported in 1957 [4]
- Several studies showed that it had a potential for abuse [5]
- Withdrawn as an anti-obesity agent in 1973
- Still largely consumed illegally as a psychostimulant
- Several cases of deaths from its misuse have been published since its withdrawal [6]

**Aminorex**

- Mechanism of action: serotonin reuptake inhibitor
- First introduced in 1962
- Became available within three years of introduction as an over-the-counter weight loss pill
- Between 1965 and 1972, there was a large increase in the incidence of primary pulmonary hypertension[7]
- The pulmonary hypertension epidemic ended in 1972 following withdrawal of aminorex from the market

**Benfluorex**

- Mechanism of action: serotonin reuptake inhibitor
- Approved in 1976 as an add-on treatment in obese patients with diabetes mellitus
- Cases of valvulopathy attributed to its use began to appear from 2003
- Withdrawn in 2009 following an epidemic of valvulopathy attributed to its use

- Several deaths reported
- To date over 3000 hospitalizations and at least 1300 deaths attributed to its use in France alone[8]

**Chlorphentermine**

- Mechanism of action: serotonin reuptake inhibitor
- Approved for obesity treatment in 1962 based on the results of short-term clinical trials [9,10]
- In 1969, animal studies began to emerge consistently suggesting an association with an increased risk of pulmonary phospholipidosis [11,12]
- Withdrawn in the same year
- No case reports of pulmonary hypertension in humans

**Clobenzorex**

- Mechanism of action: serotonin-norepinephrine-dopamine releasing agent
- Approved in 1966 as an appetite suppressant
- Cases of drug abuse and psychiatric adverse reactions appeared from 1986 [13]
- Withdrawn from the market in 2000

**Cloforex**

- Mechanism of action: serotonin reuptake inhibitor
- A prodrug of chlorphentermine (see above)
- Introduced for obesity treatment in1965
- Cases of suspected pulmonary hypertension attributed to its use reported in Germany
- Animal studies suggested an association with pulmonary vasculopathy [14]
- Withdrawn in 1967

**Dexfenfluramine**

- Mechanism of action: serotonin reuptake inhibitor
- Approved in 1995 as an anti-obesity agent
- Several cases of cardiovascular adverse reactions reported shortly after [15]
- Withdrawn within two years of approval
- Cases of pulmonary hypertension attributed to its use were reported before regulatory approval [16,17]

**Fenbutrazate**

- Mechanism of action: norepinephrine-dopamine releasing agent
- Approved for use in obesity in 1957
- Cases of drug abuse reported in France [18]
- First withdrawn in 1969, and then withdrawn in all of Europe in 1995 [19]

**Fenfluramine**

- Mechanism of action: serotonin reuptake inhibitor
- First approved in in 1973
- Reports of pulmonary hypertension first appeared in 1981
- Several other case reports subsequently published
- Epidemiological studies showed an association between fenfluramine and pulmonary hypertension [[20]]
- Withdrawn worldwide in 1997

**Fenproporex**

- Mechanism of action: norepinephrine releasing agent
- First approved as an anti-obesity drug in 1966
- Cases of drug abuse attributed to its use first reported in 1997 [[21]]
- Withdrawn in Europe and Brazil within two years of the initial reports
- A recent systematic review evidence concluded that its abuse potential and amphetamine-like adverse effects remain a cause of concern [[22]]

**Levamfetamine**

- Mechanism of action: serotonin-norepinephrine-dopamine releasing agent
- Introduced in 1944 as an anti-obesity agent
- Within 10 years of approval, cases of psychiatric reactions due to misuse were reported [[23]]
- Several cases of abuse and dependence (with psychoses) were subsequently reported [[24]]
- Withdrawn from the market in 1970

**Mazindol**

- Mechanism of action: norepinephrine-dopamine releasing agent
- Approved as an anti-obesity drug in 1970
- Neurotoxicity due to an interaction with lithium reported in 1980 [[25]]
- Several cases of testicular pain attributed to its use also reported [[26]]
- Further cases of psychiatric adverse reactions subsequently published [[27]]
- Withdrawn from the market in 1987

**Meferonex**

- Mechanism of action: serotonin-norepinephrine-dopamine releasing agent
- Approved for treatment of obesity in 1966
- Serious neuropsychiatric adverse reactions due to abuse reported in 1995
- Withdrawn in Europe four years later [[28]]

**Metamfetamine**

- Mechanism of action: serotonin-norepinephrine-dopamine releasing agent
- First approved for obesity treatment in 1944

- Cases of abuse-related suspected psychiatric adverse reactions first appeared 10 years later [29]
- Several cases of abuse resulting in neurotoxicity or cardiotoxicity subsequently reported
- By 1968 reports of deaths from its use had appeared [30]
- A further case series of harms was reported two years later [31]
- Withdrawn in 1973

**Phendimetrazine**

- Mechanism of action: Norepinephrine-dopamine releasing agent
- First approved for obesity management in 1961
- Cases of abuse were first reported in 1979 [32]
- Withdrawn in 1982

**Phenmetrazine**

- Mechanism of action: norepinephrine-dopamine releasing agent
- First approved for management of obesity in 1956
- Cases of drug abuse and addiction appeared within three years of approval [33]
- Withdrawn as an anti-obesity agent in 1982

**Phentermine**

- Mechanism of action: norepinephrine-dopamine releasing agent
- First approved in 1959
- Several cases of lung phospholipidosis in animals and humans reported thereafter
- Reports of deaths began to appear in 1974 [34]
- Withdrawn from most countries where it was marketed in 1981
- Still available for short-term management of obesity in the USA

**Phenylpropanolamine (norpseudoephedrine)**

- Mechanism of action: norepinephrine-dopamine releasing agent
- Initially used as a nasal decongestant
- Introduced in 1947 for obesity
- Cases of psychosis attributed to its use began to appear in 1966 [35]
- Cases of intracranial haemorrhage reported in 1985 [36]
- Withdrawn within two years of the reports of haemorrhages

**Pipradrol**

- Mechanism of action: norepinephrine-dopamine reuptake inhibitor
- Approved as an adjunct to dietary management of obesity in 1953
- Associated with several cases of drug abuse [37]
- Withdrawn as an anti-obesity agent in 1982
- Still available for management of ADHD and narcolepsy

**Pyrovalerone**

- Mechanism of action: norepinephrine-dopamine releasing agent
- Introduced as a weight loss drug in 1974
- Within a year of introduction, cases of abuse started to appear [38]
- Withdrawn as an anti-obesity 1979 owing to problems associated with abuse and dependence
- A derivative, methylenedioxypyrovalerone (MDPV), is marketed illegally as a designer recreational drug under the name of “Bath Salts” [39]

**Rimonabant**

- Mechanism of action: cannabis $CB_1$ receptor antagonist/inverse agonist
- Approved in Europe in 2006 for obesity treatment
- Within 1 year of approval, cases of severe psychiatric adverse reactions were reported [40]
- 5 deaths attributed to its use in the UK
- Withdrawn in 2007

**Sibutramine**

- Mechanism of action: serotonin-norepinephrine reuptake inhibitor
- Approved in 1997 in the USA and Europe in 2001
- Within a year of its European approval, serious cardiovascular adverse reactions were reported, resulting in temporary withdrawal in Italy [41]
- Several cases of severe cardiovascular adverse reactions, including deaths, subsequently reported [42]
- Withdrawn in Europe and the USA in 2010

# REFERENCES


1 Rigo P, Collignon P, Booz J. A propos d'un cas de syndrome de pickwick. [A case of Pickwickian syndrome.] Acta Clin Belg 1974; 29(3): 168-75.

2 Thomas SH, Butt AY, Corris PA, Egan JJ, Higenbottam TW, Madden BP, Waller PC. Appetite suppressants and primary pulmonary hypertension in the United Kingdom. Br Heart J 1995; 74(6): 660-3.

3 Lesses MF, Myerson A. Human autonomic pharmacology. XVI. Benzedrine sulfate as an aid in the treatment of obesity. N Engl J Med 1938; 218: 119-124.

4 Simpson WS. Toxic psychosis; a complication of overdosage of anti-obesity drugs. J Kans Med Soc 1957; 58(8): 524-7.

5 Johnson J, Milner G. Johnson J, Milner G. Amphetamine intoxication and dependence in admissions to a psychiatric unit. Br J Psychiatry 1966; 112(487): 617-9.

6 Kalant H, Kalant OJ. Death in amphetamine users: causes and rates. Can Med Assoc J 1975; 112(3): 299-304.

7 Fishman AP. Aminorex to fen/phen: an epidemic foretold. Circulation 1999; 99(1): 156-61.

8 Fournier A, Zureik M. Estimate of deaths due to valvular insufficiency attributable to the use of benfluorex in France. Pharmacoepidemiol Drug Saf 2012; 21(4): 343-51.

9 Levin J, Trafford JAP, Newland PM, Bishop PMF. Practitioner 1963; 191: 65.

10 Seaton DA, Rose K, Duncan LJP. Practitioner 1964; 193: 698.

11 Franken G, Lüllmann H, Siegfriedt A. Uber ein Massenhaftes Auftreten von "Schaumzellen" in Rattenlungen nach chronischer Gabe von Chlorphentermin. [A massive appearance of "foam cells" in rat lungs following chronic administration of chlorphentermine.] Naunyn Schmiedebergs Arch Pharmakol 1970; 266(4): 323-4.

12 Parwaresch R, Reil GH, Seiler KU. Uber die Tier- und Organspezifität morphologischer Veranderungen nach chronischer Chlorphentermingabe. [Studies on animal and organ specificity of chlorphentermine induced morphological alterations.] Res Exp Med (Berl) 1973; 161(4): 272-88.

13 Cornaert P, Camblin J, Graux P, Anaye B, Dutoit A, Croccel L. Cardiomyopathie congestive au cours d'une toxicomanie à un anorexigène, le clobenzorex. [Congestive cardiomyopathy in addiction to clobenzorex, an anorexigenic drug.] Arch Mal Coeur Vaiss 1986; 79(4): 515-8.

14 Magnusson G, Magnusson O. Cloforex-induced pulmonary changes in rats. Beitr Pathol 1972; 146(1): 79-88.

15 Cacoub P, Dorent R, Nataf P, Houppe JP, Piette JC, Godeau P, Gandjbakhch I. Pulmonary hypertension and dexfenfluramine. Eur J Clin Pharmacol 1995; 48(1): 81-3.

16 Ferrari E, Drai E, Jourdan J, Sanchez B, Baudouy M, Morand P. Hypertension artérielle pulmonaire sévère compliquant un long traitement par la dexfenfluramine. [Severe pulmonary hypertension complicating a long treatment with dexfenfluramine.] Arch Mal Coeur Vaiss 1994; 87(2): 285-6.

17 Douglas JG, Munro JF, Kitchin AH, Muir AL, Proudfoot AT. Pulmonary hypertension and fenfluramine. Br Med J (Clin Res Ed) 1981; 283(6296): 881-3.

18 World Health Organization. WHO Expert Committee on Drug Dependence. http://apps.who.int/iris/bitstream/10665/39635/1/WHO_TRS_729.pdf [Accessed 1st August, 2016].

19 European Commission. List of the names of the medicinal products, marketing authorisation holders, pharmaceutical forms, strengths, route of administration, packaging, and package sizes in the member states. http: //ec.europa.eu/health/documents/community-register/2000/200003093430/anx_3430_en.pdf [Last accessed 1st August, 2016].

20 Connolly HM, Crary JL, McGoon MD, Hensrud DD, Edwards BS, Edwards WD, Schaff HV. Valvular heart disease associated with fenfluramine-phentermine. N Engl J Med. 1997 Aug 28;337(9):581-8.

21 Silva OA, Yonamine M, Antunes CLG, Greve JMD, Midio AF. Fenproporex abuse by truck drivers in Brazil. Presented at the 1998 Joint SOFT-TIAFT International meeting, Albuquerque, NM. 1998.

22 Paumgartten FJ, Pereira SS, de Oliveira AC. Safety and efficacy of fenproporex for obesity treatment: a systematic review. Rev Saude Publica 2016; 50: 25.

23 Schinko H, Solms W. Eine Psychose bei Adipexsuchtigkeit. [A psychosis in adipex addiction.] Wien Z Nervenheilkd Grenzgeb 1954; 9(3): 290-301.

24 Simpson WS. Toxic psychosis; a complication of overdosage of anti-obesity drugs. J Kans Med Soc 1957; 58(8): 524-7.

25 Hendy MS, Dove AF, Arblaster PG. Mazindol-induced lithium toxicity. Br Med J 1980; 280(6215): 684-5.

26 McEwen J, Meyboom RH. Testicular pain caused by mazindol. Br Med J (Clin Res Ed) 1983; 287(6407): 1763-4.

27 Rihmer Z, Révai K, Arató M, Perényi A. Two case reports of mazindol-induced depression. Am J Psychiatry 1984; 141(11): 1497-8.

28 World Health Organization. Anorectic agents – withdrawal of marketing authorizations recommended: EC, Portugal. WHO Pharmac Newslett 1999, No. 09&12.

29 Carr RB. Acute psychotic reaction after inhaling methylamphetamine. Br Med J 1954; 1(4877): 1476.

30 Cravey RH, Baselt RC. Methamphetamine poisoning. J Forensic Sci Soc 1968; 8(2): 118-20.

31 Smith DE, Fischer CM. An analysis of 310 cases of acute high-dose methamphetamine toxicity in Haight-Ashbury. Clin Toxicol 1970; 3(1): 117-24.

32 Jain NC, Budd RD, Sneath TC. Frequency of use or abuse of amphetamine-related drugs. Am J Drug Alcohol Abuse. 1979;6(1):53-7.

33 Evans J. Psychosis and addiction to phenmetrazine (Preludin). Lancet 1959; 2(7095): 152-5.

34 Price K. "Case Notes: Phentermine," Bull Int Assoc Foren Toxicol 1974; 10(1): 12.

35 Kane FJ Jr, Green BQ. Psychotic episodes associated with the use of common proprietary decongestants. Am J Psychiatry 1966; 123(4): 484-7.

36 Kikta DG, Devereaux MW, Chandar K. Intracranial hemorrhages due to phenylpropanolamine. Stroke 1985; 16(3): 510-2.

37 Goldberg L. Drug abuse in Sweden. Bull Narcot 1968; 1: 1-31. http: //www.lycaeum.org/research/researchpdfs/1722.pdf [Last accessed 20th July, 2016].

38 Deniker P, Lôo H, Cuche H, Roux JM. Utilisation abusive par les toxicomanes d'un psycho-stimulant, la pyrovalérone [Abuse of pyrovalerone by drug addicts.] Ann Med Psychol (Paris) 1975; 2(4): 745-8.

39 Prosser JM, Nelson LS. The toxicology of bath salts: a review of synthetic cathinones. J Med Toxicol. 2012; 8(1): 33-42.

40 Gadde KM. Effect of rimonabant on weight and cardiometabolic risk factors. JAMA. 2006 Aug 9;296(6):649-50; author reply 650-1.

41 Anonymous. Italy suspends sibutramine licence. Pharm J 2002; 268(7190): 385-391.

42 Wooltorton E. Obesity drug sibutramine (Meridia): hypertension and cardiac arrhythmias. CMAJ. 2002;166(10):1307-8.
