# Supplementary material for: Post-marketing withdrawal of anti-obesity medicinal products because of adverse drug reactions: a systematic review
Source: BMC Med. 2016 Nov 29;14:191. doi: 10.1186/s12916-016-0735-y (PMC5126837; doi:10.1186/s12916-016-0735-y)
Supplement: Additional file 4: — Publication trends* for scientific articles related to obesity prevalence and treatments over time. (PDF 16 kb) [file 12916_2016_735_MOESM4_ESM.pdf]

Web appendix 4: Publication trends* for scientific articles related to obesity prevalence and treatments over time

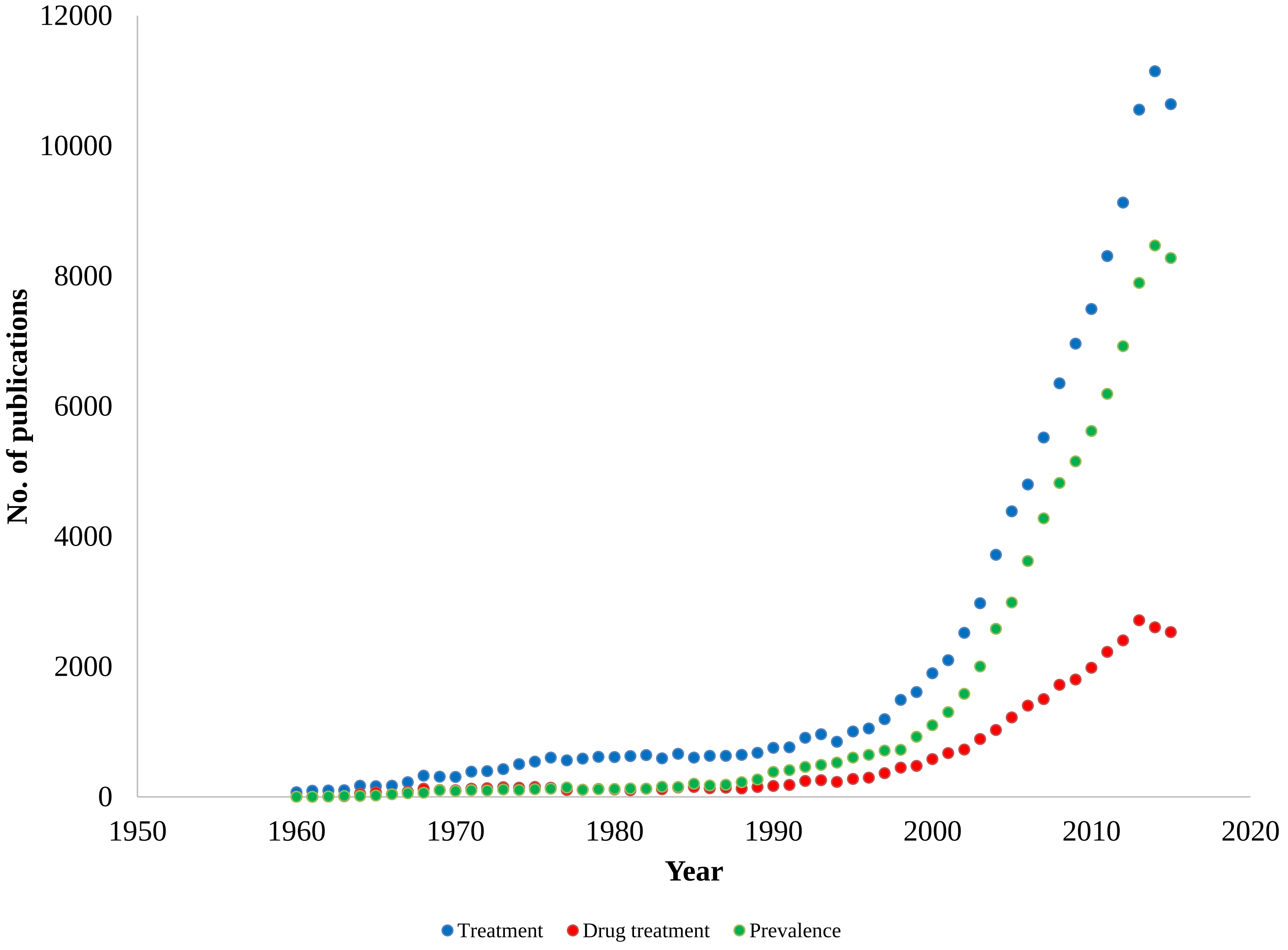

*Data sourced using PubMed trends
